# Development of a chitosan‐modified PLGA nanoparticle vaccine for protection against *Escherichia coli* K1 caused meningitis in mice

**DOI:** 10.1186/s12951-021-00812-9

**Published:** 2021-03-05

**Authors:** Jin Zhang, Hongwu Sun, Chen Gao, Ying Wang, Xin Cheng, Yun Yang, Qiang Gou, Langhuang Lei, Yanping Chen, Xingyong Wang, Quanming Zou, Jiang Gu

**Affiliations:** 1 Department of Respiratory, Hunan Children’s Hospital, Changsha, China; 2 Department of Pediatric Research Institute, Children’s Hospital of Chongqing Medical University, Chongqing, China; 3 National Engineering Research Center of Immunological Products, Department of Microbiology and Biochemical Pharmacy, College of Pharmacy, Third Military Medical University, The 30th, Gaotanyan Street, Shapingba District, Chongqing, 400038 China

**Keywords:** *Escherichia coli* K1, Chitosan, PLGA, Nanoparticles, Vaccine

## Abstract

**Background:**

*Escherichia coli* K1 (*E. coli* K1) caused neonatal meningitis remains a problem, which rises the urgent need for an effective vaccine. Previously, we rationally designed and produced the recombinant protein OmpAVac (Vo), which elicited protective immunity against *E. coli* K1 infection. However, Vo has limited stability, which hinders its future industrial application.

**Method:**

Chitosan-modified poly (lactic-*co*-glycolic acid) (PLGA) nanoparticles were prepared and used as carried for the recombinant Vo. And the safety, stability and immunogenicity of Vo delivered by chitosan-modified PLGA nanoparticles were tested in vitro and in a mouse model of bacteremia.

**Results:**

We successfully generated chitosan-modified PLGA nanoparticles for the delivery of recombinant Vo (VoNP). In addition, we found that a freeze-drying procedure increases the stability of the VoNPs without changing the shape, size distribution and encapsulation of the Vo protein. Unlike aluminum adjuvant, the nanoparticles that delivered Vo were immunoprotective in mice even after storage for as long as 180 days.

**Conclusions:**

We identified an effective strategy to improve the stability of Vo to maintain its immunogenicity, which will contribute to the future development of vaccines against *E. coli* K1.
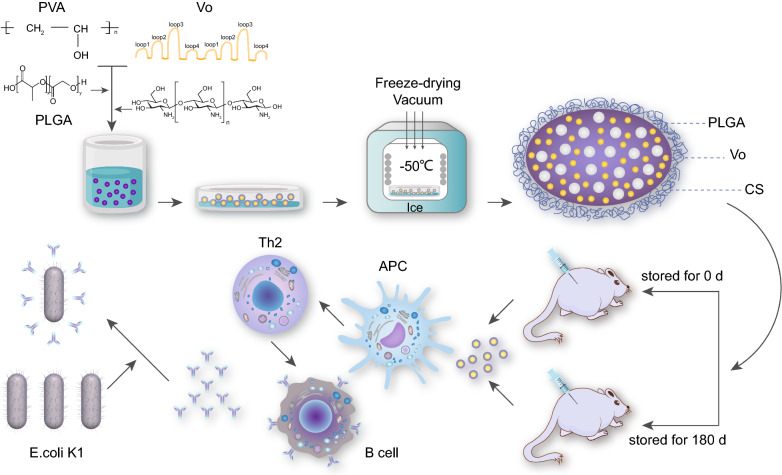

## Background

Neonatal bacterial meningitis (NBM) is a rapidly developing and life-threatening disease. Although advances in chemotherapies and supportive care have reduced the number of deaths, up to 50% of survivors show long-term disability and severe neurological dysfunction [1, 2]. Globally, the *Escherichia coli* strain carrying the K1 capsule (*E. coli* K1) is the most common cause of NBM in early neonates and has a high mortality rate [3]. Even with the application of effective third-generation antibiotics, the morbidity and mortality rates associated with *E. coli* K1 meningitis have remained unchanged in the last few decades [4]. Hence, the development of alternative strategies for the control of *E. coli* K1-induced meningitis is urgently needed. Of note, many vaccine candidates have been tested, but no vaccine has been approved to date [1].

The outer membrane protein A (OmpA), a highly conserved and abundant outer membrane protein of *E. coli* K1, is essential for maintaining the integrity of the outer membrane and in bacterial conjugation [5]. In addition, the bacteria then use OmpA to bind Ecgp96 on the blood–brain barrier to invade the brain endothelium and disseminate into the central nervous system. In this process, OmpA disrupts the tight junctions between BMECs, leading to increased permeability [6, 7]. For the reasons above, OmpA has become a critical target of vaccines against *E. coli* K1 in recent years [8, 9].

The N-terminal membrane-anchoring domain of OmpA has eight transmembrane β-strands, which are connected by three periplasmic turns and four relatively long surface-exposed hydrophilic loops [10]. The C-terminal domain interacts with the peptidoglycan layer in the periplasm to maintain outer membrane integrity [11]. Based on the structure of OmpA, we rationally designed and successfully generated the artificial protein OmpAVac (Vo), which is composed of the surface loops from OmpA. Vaccination of Vo formulated with aluminum hydroxide induced Th1, Th2, and Th17 immune responses and conferred adequate protection in mice [12]. However, the recombinant Vo protein is not stable in vitro and in vivo, which hinders its further industrial application.

Poly (lactic-*co*-glycolic acid) (PLGA)-based systems are a promising vehicle for vaccines because PLGA nanoparticles could simplify vaccination programs, enhance efficiency, and increase the immunogenicity of the encapsulated antigens [13, 14]. Additionally, due to its biodegradable and biocompatible characteristics, PLGA has been applied in drug delivery, medical treatment, and tissue engineering by the FDA [15]. Moreover, FDA-approved chitosan is another promising carrier for the delivery of vaccine antigens. Chitosan (CS) is cheap and compatible with a wide range of drugs. Furthermore, it can easily interact with the cell membrane due to its positive charge, which results in its high formability and biodegradability [16]. For these reasons, we hypothesize that a carrier based on PLGA and CS nanoparticles could improve the stability and bioavailability of Vo.

In this study, chitosan-modified PLGA nanoparticles were produced and used as carriers of Vo protein. The safety, stability and immunogenicity of Vo formulated with the chitosan-modified PLGA nanoparticles (VoNPs) were tested in vitro and in a mouse model of bacteremia.

## Methods

### Ethical consideration

All experiments complied with ethical regulations and were approved by the Animal Ethical and Experimental Committee of the Third Military Medical University (No. TMMU0157).

### Mice and strains

Six- to eight-week-old specific pathogen-free female BALB/c mice were purchased from Beijing HFK Bioscience Limited Company (Beijing, People’s Republic of China). The mice were maintained under barrier conditions in a biohazard animal room. The *E. coli* K1 strain RS218 was obtained from the ATCC (No. 700973).

### Formulation of chitosan‐coated PLGA nanoparticles

We tested five formulations to determine the optimal formula for the nanoparticles. The formulations included chitosan (CS) nanoparticles (S1), poly(lactic-*co*-glycolic) acid (PLGA) nanoparticles with dichloromethane as the solvent (S2), PLGA nanoparticles with acetone as the solvent (S3), CS nanoparticles modified with PLGA (S4), and PLGA nanoparticles modified with CS (S5). To produce CS nanoparticles (S1), CS was dissolved in 1% aqueous acetic acid. Then, sodium polyphosphate (1 mg/ml) was added into the CS solution drop by drop and stirred at 800 rpm for 8 h. To produce PLGA nanoparticles, PLGA was dissolved in dichloromethane (S2) or 1% aqueous acetone (S3). Then, 2% of poly vinyl alcohol (PVA) was added drop by drop under magnetic stirring at 800 rpm for 8 h. The nanoparticles were washed three times with deionized water and resuspended for further use. To produce the CS nanoparticles modified with PLGA (S4), CS nanoparticles were prepared as described for S1, and then the PLGA dissolved in dichloromethane was added into the CS solution and stirred at 800 rpm for an additional 8 h. To produce the PLGA nanoparticles modified with CS (S5), the PLGA nanoparticles were prepared as described for S3, and then the CS dissolved in 1% aqueous acetic acid was added and stirred at 800 rpm for an additional 8 h.

To prepare CS-PLGA nanoparticles carrying Vo (VoNPs), the recombinant Vo protein was produced as described previously [12]. Then, the purified Vo was added into 2% PVA buffer. The VoNPs were then prepared as described for S5. The blank CS-PLGA nanoparticles (BNPs) were produced as described for S5. The freeze-drying cycle involved freezing at − 50°C, followed by the sublimation of frozen water at 0.050 M bar until complete dryness after 25 h. The dried samples were stored at 4 °C.

### Analysis of morphology and stability

Characterization of the nanoparticles was carried out with scanning electron microscopy (Zeiss, Germany) and transmission electron microscopy (JEOL, Japan). The three-dimensional structure was measured by atomic force microscopy (ATM, CU, China). The size, polydispersity index (PDI) and zeta potential were determined by a Nano ZS (Zetasizer 3000 HAS, Malvern Instrument, England). A MALDI-TOF mass spectrometer (Shimadzu, Japan) was used to evaluate the stability of the proteins in the nanoparticles. The entrapment efficiency was calculated according to the following formulas: Entrapment efficiency (%) = (Amount_total Vo_ − Amount_loading Vo_)/Amount_total Vo _× 100.

### Evaluation of the cytotoxicity of VoNPs

The L929 cells were grown in Dulbecco’s Modified Eagle’s Medium (DMEM) containing 10% fetal bovine serum (FBS). When the cells reached approximately 70–80% confluence, the cells were washed with fresh DMEM and transferred to fresh 96-well plates at a density of 5 × 10^3^ per well. Then, different concentrations of VoNPs and BNPs were added and incubated with the cells at 37 °C for 48 h. The same volumes of DMEM medium and DMSO were used as negative and vehicle controls, respectively. The cell viability was determined using a commercial CCK-8 assay kit following the manufacturer’s instructions.

### Characterization of protein release from VoNPs

The release of Vo from VoNPs was evaluated in PBS (pH = 6.8) at 37 °C. Briefly, a sample comprising 1 ml of the VoNPs suspension (1.0 mg/ml) was placed in a dialysis bag (cut-off = 10 kDa, Sangon, Shanghai, China) and dialyzed against 5 ml of PBS. Samples comprising 50 µl of the dialysate were collected at 0.25, 0.5, 1, 2, 4, 8, 12, 24, 48, 72, 96, 120 and 144 h. The concentration of Vo was detected using a Bradford assay and the protein release ratio was calculated. To characterize the release of protein from nanoparticles in vivo, green fluorescent protein (GFP) was encapsulated in nanoparticles (GFP-NPs). After anesthesia, nude mice were administered an intramuscular injection comprising 500 µl of the GFP-NPs or GFP in PBS buffer (25 µg/per mouse). Then, the nude mice were scanned using an IVIS system (Caliper Life Sciences, MA, USA) at 0.5, 1,2, 4, 8, 12, 24, 36 and 48 h after the administration. A background scan was also recorded immediately before the intramuscular administered to provide a threshold for adjusting the images collected at later time points.

### Mouse immunization and challenge

In each group, mice were intramuscularly immunized with 500 µl of vaccine on days 0, 7, and 14. Each 500 µl of vaccine contained 25 µg of Vo or Vo with 500 µg of aluminum adjuvant. Immunizations with blank nanoparticles, aluminum adjuvant and PBS alone were performed as negative controls. The mice were sacrificed on day 21, and their sera were collected to determine the titer of antigen-specific antibodies.

Mice were intraperitoneally injected with a lethal dose (1 × 10^8^ CFU per mouse) of *E. coli* K1 RS218 in 100 µl of sterilized PBS. The number of deaths was recorded daily for 5 days. For further analysis of the protective mechanism, another five mice were intraperitoneally challenged with a sublethal dose (1 × 10^7^ CFU per mouse) of *E. coli* K1. Their body weights were monitored daily for 5 days, and the percentage of the initial weight was calculated. In addition, five immunized mice in each group were sacrificed at 24 h after the challenge, and the bacterial loads in their blood and spleens were determined. The spleens of the sacrificed mice were collected, weighed, and homogenized in 1 ml of sterilized PBS buffer. The spleen homogenates and blood were plated onto LB plates at a tenfold serial dilution and cultured at 37 °C for 20 h. The number of colonies on the plates was counted and used to calculate the bacterial load. The CFU per gram of tissue was calculated for the comparison of bacterial load.

### ELISA

ELISA was used to evaluate the humoral immune response elicited by vaccination. In brief, 96-well ELISA plates were coated with purified Vo (3 µg/ml) and incubated overnight at 4 °C. Then, the antigen-coated plates were blocked with 200 µl of blocking buffer [PBS containing 0.01% Tween 20 and 2% BSA, pH 7.5 (PBST)] overnight at 4 °C. Serially diluted (twofold) sera (100 µl) were then added to each well, followed by incubation for 60 min at 37 °C. After three rinses with PBST, the plates were incubated with HRP-conjugated anti-mouse Fc antibodies (Abcam) for 45 min at 37 °C. Finally, the color was developed with TMB (Sigma), and the reaction was stopped by adding 0.1 M sulfuric acid. The optical density (OD) was determined at 450 nm. The subtype of anti-Vo IgG was also determined via ELISAs.

### Characterization of in vitro protection by anti-VoNP antibodies

Firstly, the opsonophagocytic killing activity of sera from VoNP-immunized mice was determined as described by Gu et al. [12]. Briefly, HL-60 cells (ATCC CCL-240) were grown and differentiated into granulocyte-like cells. Then 10^3^ CFU of *E. coli* K1, diluted serum, and HL-60 cells were added to 96-well plates. After the introduction of complement components (1% infant rabbit serum), the mixtures were co-incubated at 37 °C for 3 h. Then, the mixtures were plated onto agar medium and incubated for 10 h. The number of bacterial colonies on the plates was counted, and the percentage of killed bacteria was calculated to evaluate the bactericidal activity using the formula: (N_PBS_ − N_serum_)/N_PBS_×100%, where N_PBS_ and N_serum_ are the numbers of bacteria in cells treated with PBS and serum, respectively.

Secondly, the effects of anti-VoNP antibodies on bacterial attachment and invasion were accessed as described previously [12]. Briefly, 10^7^ CFU of *E. coli* K1 RS218 was incubated with 3 mg/ml of anti-VoNP, -Vo + Al or BNP antibodies for 1 h on ice, and then used to infect BMECs (CD31^+^) at a ratio of 1:100. After incubation at 37 °C for 2 h, the cells were washed with M199 medium to remove the unattached bacteria. To quantify the cell-associated bacteria, cells were treated with 0.5% Triton X-100 in PBS and plated onto LB agar. To quantify the invading bacteria, the cells were treated with gentamicin (100 µg/ml) for 1 h to kill bacteria that remained outside the cells. Then, bacteria were released from the cells using 0.5% Triton X-100 and enumerated by plating onto LB agar.

### Statistical analysis

The data are presented as the mean ± SE (standard error). The significance of the differences was determined by unpaired parametric tests (Student’s t-test for two groups or one-way ANOVA for three or more groups). The significance of the differences in bacterial load was determined by unpaired nonparametric tests (Mann–Whitney test). The Kaplan–Meier test was employed for analysis of the survival rate. GraphPad Prism 6 was used for data analyses. Differences were considered significant when the *P* value was < 0.05. All experiments, except for animal challenge assays, were conducted a minimum of three times.

## Results

### Chitosan‐modified PLGA nanoparticles are suitable for preparing VoNPs

**Fig. 1:**
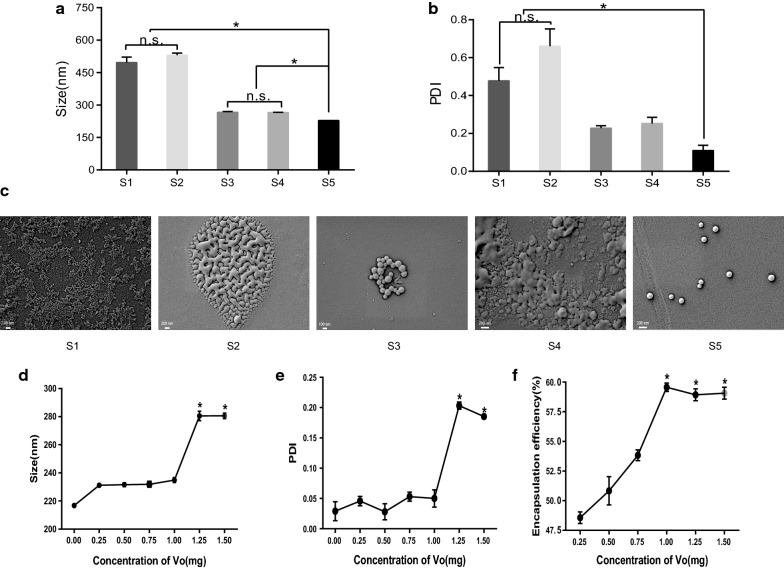
Chitosan-modified PLGA nanoparticles are suitable for the formulation of Vo nanoparticles. **a** The size distribution of nanoparticles from five formulations. S1: Chitosan nanoparticles; S2: PLGA nanoparticles with dichloromethane as a solvent; S3: PLGA nanoparticles with acetone as a solvent; S4: Chitosan nanoparticles modified with PLGA; S5: PLGA nanoparticles modified with chitosan (CS-PLGA). **b** The polydispersity index (PDI) of nanoparticles from the five formulations. **c** Scanning electron microscopy observation of nanoparticles from the five formulations. **d** The size distribution of CS-PLGA nanoparticles loaded with a gradient of recombinant Vo. **e** The PDI of CS-PLGA nanoparticles loaded with a gradient of concentrations of recombinant Vo. **f** Entrapment efficiency of CS-PLGA nanoparticles loaded with a gradient of concentrations of recombinant Vo. *P < 0.05

To determine the optimal formula of nanoparticles, we designed five formulations: CS nanoparticles (S1); PLGA nanoparticles with dichloromethane as the solvent (S2); PLGA nanoparticles with acetone as the solvent (S3); CS nanoparticles modified with PLGA (S4); and PLGA nanoparticle modified with CS (CS-PLGA, S5). The results showed that the particle sizes of S1, S2, S3, S4 and S5 were 496.7 ± 42.6 nm, 530.3 ± 17.2 nm, 266.3 ± 6.4 nm, 265.2 ± 2.1 nm, and 228.5 ± 0.2 nm, respectively. There was no significant difference in the particle size among S1 and S2 or S3 and S4 (*P* > 0.05). The particle size of S5 was significantly smaller than that of the other formulations (*P* < 0.05) (Fig. 1a). The polydispersity index (PDI) values of S1, S2, S3, S4 and S5 were 0.4777 ± 0.1209, 0.6600 ± 0.1587, 0.2277 ± 0.0228, 0.2520 ± 0.057, and 0.1097 ± 0.0486, respectively. Similar to the trend of particle size, the PDI of S5 was significantly lower than that of S1 and S2 (*P* < 0.05) (Fig. 1b). Scanning electron microscopy results showed that the nanoparticles from S5 had a regular spherical shape, good dispersion and no aggregation (Fig. 1c). Consistent with the DLS observations, the average size of the particles was also approximately 200 nm when determined by SEM (Fig. 1c).

Next, a gradient of concentrations of recombinant Vo was formulated with the CS-PLGA nanoparticles to determine the maximal encapsulation. Clearly, the particle size of the nanoparticles increased with the increase in loaded Vo. No significant difference in the particle sizes was observed when the amount of Vo was lower than 1.25 mg. However, when the amount of Vo reached 1.25 mg and 1.50 mg, the particle size of the VoNPs increased sharply (*P* < 0. 05) (Fig. 1d). A similar trend was observed for the polydispersity index (PDI) (Fig. 1e). The PDI of the VoNPs remained between 0.02 and 0.05 when the amount of Vo protein was 1.0 mg or less, and no significant difference was observed among the groups (*P* > 0.05). Nevertheless, when the amount of Vo protein was up to 1.25 mg and 1.50 mg, the PDI increased dramatically (*P* < 0. 05), which indicates that these VoNPs had poor dispersibility and easily aggregated (Fig. 1e). In addition, the entrapment efficiency increased with increasing Vo protein and reached a peak (59.3%) at 1.0 mg of Vo (Fig. 1f), and no significant change was observed when 1.25 mg and 1.50 mg of Vo were used (*P* < 0. 05). These data suggested that the optimum amount of protein encapsulated by the VoNPs was 1.0 mg.

### Freeze‐drying did not affect the formation of the VoNPs

**Fig. 2:**
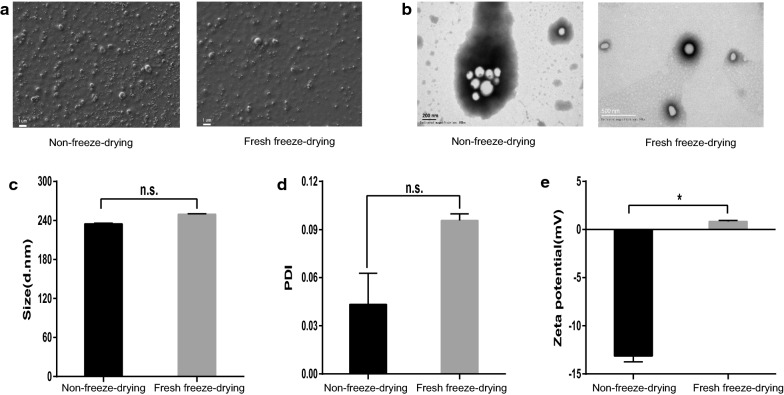
Freeze-drying did not affect the formation of the VoNPs. **a** Scanning electron microscopy observation of the VoNPs after the freeze-drying process. **b** Transmission electron microscopy observation of the VoNPs after the freeze-drying process. **c** Change of the size distribution of the VoNPs after the freeze-drying process. **d** Change of the PDI of the VoNPs after the freeze-drying process. **e** Change of the zeta potential of the VoNPs after the freeze-drying process. *n.s.* no significant difference; *P < 0.05

Freeze-drying is an effective strategy to improve the stability of nanoparticles [17]; thus, we tested whether the formulation of the VoNPs was affected after freeze-drying. The results from scanning electron microscope (SEM) and transmission electron microscopy (TEM) showed that the freeze-dried VoNPs still had a regular spherical shape, which was similar to that before freeze-drying (Fig. 2a, b). Moreover, as expected, no significant difference in particle size and PDI was observed between the freeze-dried and non-freeze-dried VoNPs (Fig. 2c, d). Interestingly, freeze-drying resulted in a significant change in the zeta potential, which increased from − 13.13 ± 0.60 mV to 0.6110 ± 0.0267 mV (*P* < 0. 05) (Fig. 2e).

### Characterization of the VoNPs

**Fig. 3:**
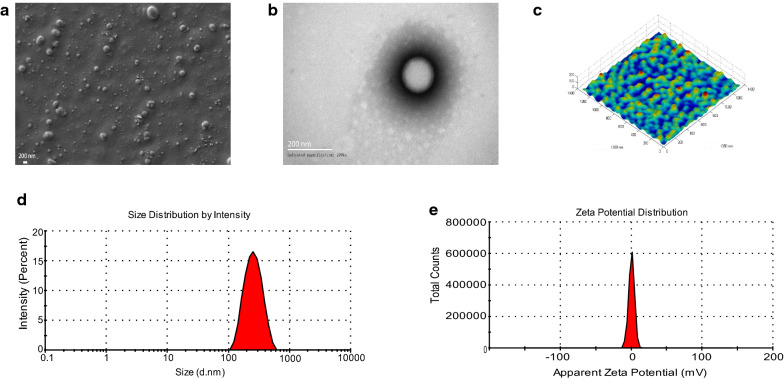
Characterization of the VoNPs. **a** Scanning electron microscopy observation of the VoNPs. **b** Transmission electron microscopy observation of the VoNPs. **c** Atomic force microscopy observation of the VoNPs. **d** The size distribution of the VoNPs. **e** The distribution of the zeta potential of the VoNPs. The VoNPs presented as uniformly smooth spherical particles with good dispersion

The VoNPs form symmetric spheres without any aggregation when viewed by TEM and SEM (Fig. 3a, b). Consistently, the AFM results showed that the VoNPs were spherical with a smooth surface, and the average size of the VoNPs was approximately 200 to 300 nm (Fig. 3c). Dynamic light scattering (DLS) showed that the size of the VoNPs was mainly between 200 and 300 nm with an average of 250.8 ± 2.13 nm and a symmetrical unimodal peak (Fig. 3d). Moreover, the distribution of the zeta potential of the VoNPs was homogeneous, with an average value of 0.6110 ± 0.0267 mV (Fig. 3e). The above results indicated that the VoNPs were spherical structures with good dispersion and smooth uniformity.

### The freeze-dried VoNPs were stable for at least 180 days

**Fig. 4:**
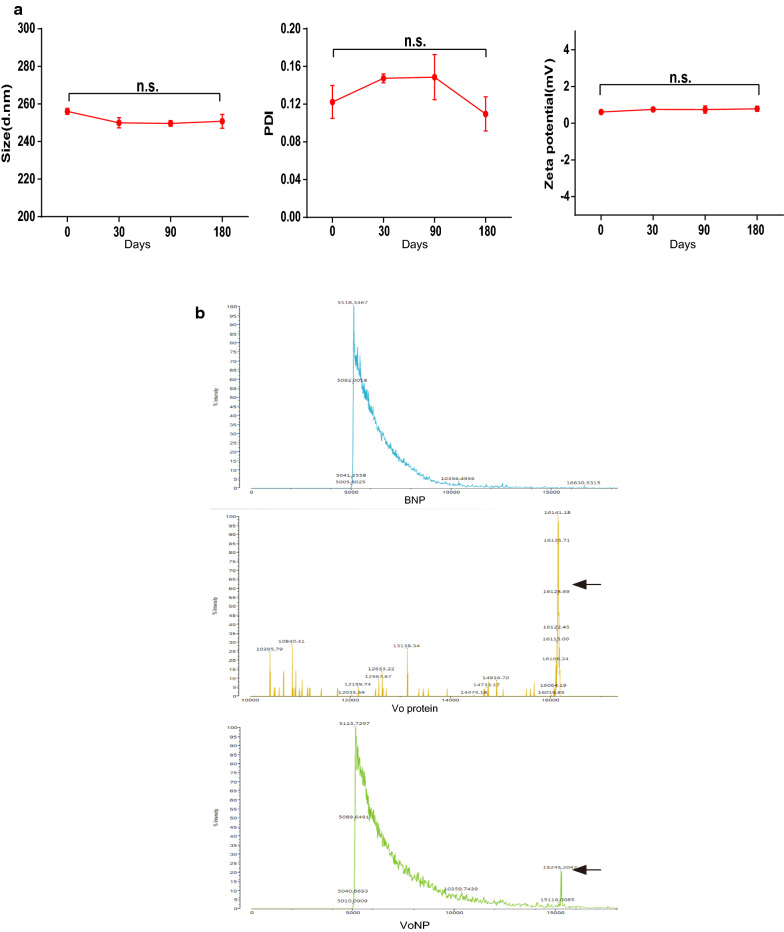
The freeze-dried VoNPs were stable for at least 180 days. **a** Changes of particle size, PDI and zeta potential (from left to right) of the VoNPs after storage at 4 °C for 30, 90 and 180 days. *n.s.* no significant difference; *P < 0.05. **b** MALDI-TOF mass spectrometry of blank nanoparticles (BNPs), Vo protein, and the VoNPs after storage at 4 °C for 180 days. The arrow indicates the Vo protein with an m/z of approximately 15,245

To evaluate the long-term stability of the VoNPs, we stored the prepared VoNPs at 4 °C and characterized them on days 0, 30, 90 and 180. The results showed that there was no significant change in particle size, PDI or zeta potential in the observation periods (*P* > 0.05) (Fig. 4a). Next, to further evaluate the stability of the protein in the VoNPs, we performed MALDI-TOF mass spectrometry to analyze the Vo protein. As shown in Fig. 4b, BNP mass spectrometry showed a special peak for the polymer. The Vo protein spectrum showed an obvious peak at approximately m/z = 15,245. In addition to the polymer peak, there was a visible ion peak at approximately m/z = 15,245 in the spectrum of the VoNPs, of which the position is the same as the peak of Vo protein alone. The above results indicated that Vo protein was still stable in the VoNPs even after 180 days of storage.

### The VoNPs were nontoxic to L929 cells and mice

**Fig. 5:**
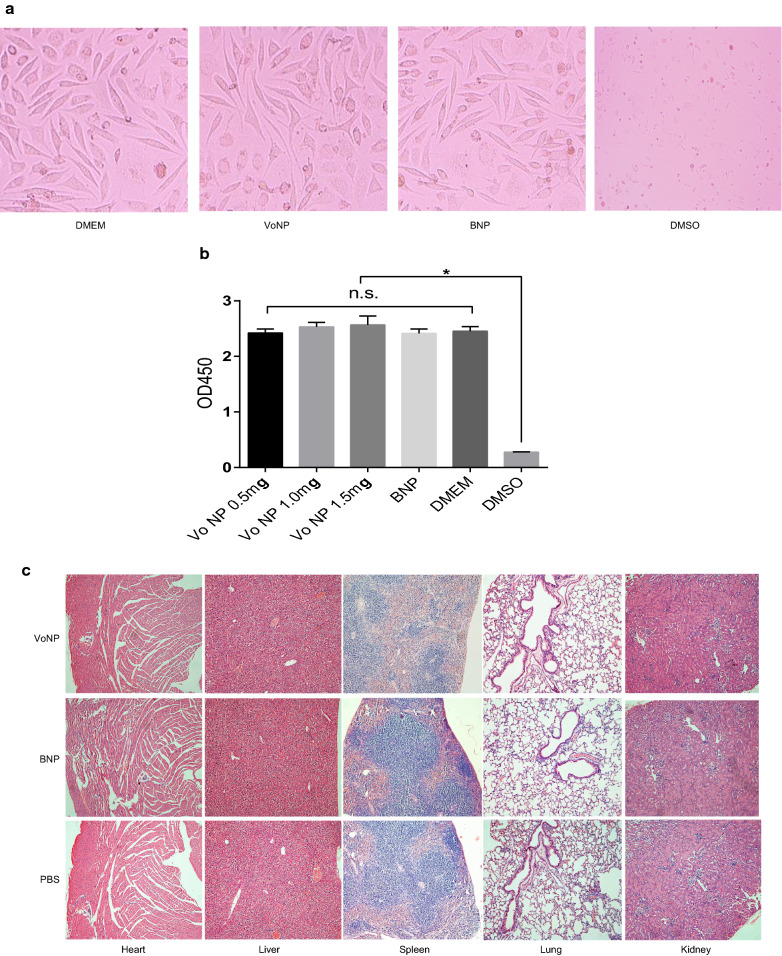
The VoNPs were nontoxic to L929 cells and mice. **a** Morphological changes in the L929 cells cultured in the presence of the VoNPs or blank nanoparticles (BNPs). **b** The optical density of cells cultured in the presence of VoNPs or BNPs. *n.s.* no significant difference; *P < 0.05. **c** Histological changes in the heart, liver, spleen, lungs and kidneys of mice injected with the VoNPs or BNPs

Safety is a crucial feature for the development of novel vaccines and delivery vectors. Therefore, the safety of the VoNPs was evaluated in vitro and in vivo. First, L929 cells were cocultured with a gradient concentration of VoNPs to evaluate cytotoxicity. No significant morphological difference was observed between the cells treated with VoNP and those treated with DMEM (Fig. 5a). The results from viability tests were consistent with the microscopic observations. Namely, there was no significant difference in the optic density among the DMEM- and all VoNP-treated cells (*P* > 0.05) (Fig. 5b). Furthermore, VoNP was intramuscularly injected into mice to evaluate its in vivo toxicity. During the observation period, no deaths or other adverse reactions occurred in the immunized mice. In addition, no significant histological change was observed in the VoNP-injected mice compared with the BNP (blank nanoparticle)- and PBS-injected mice (Fig. 5c). Collectively, these results showed that the VoNPs had no apparent toxic effect on L929 cells and mice.

### Protein release in VoNP

**Fig. 6:**
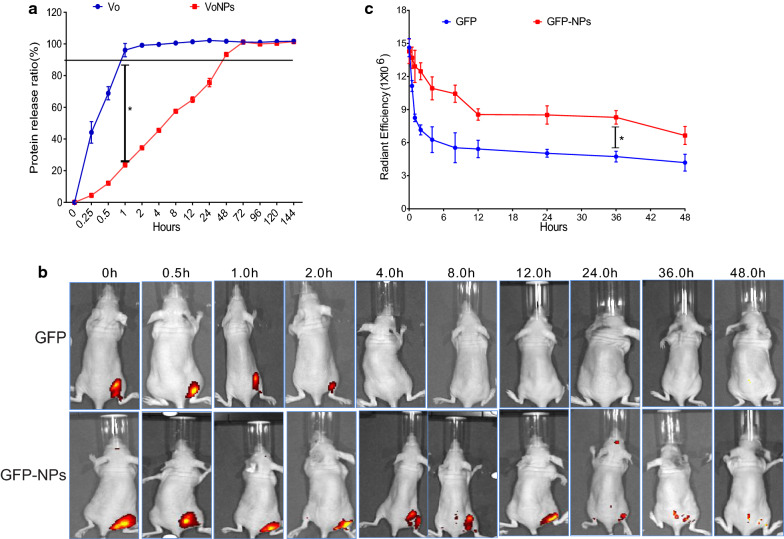
**a** In vitro protein release of Vo from VoNPs. The bars represent the means ±  standard error of the release ratio for three samples. **b** Nude mice were intramuscularly administrated green fluorescent protein (GFP) or GFP encapsulated in nanoparticles (GFP-NP). The mice were sequentially imaged from 0.5 to 48 h using an IVIS spectrum imaging system. **c** Quantitation of fluorescence intensity from the IVIS system. The data are presented as the means ±  standard error (n = 3). *P < 0.05

The in vitro release profile of Vo and the VoNPs was recorded using a Bradford assay (Fig. 6a). The release of Vo alone was obviously faster than that of VoNPs, although the release ratios of both groups reached nearly 100% in 72 h. The cumulative release of Vo alone in 1 h was 96.11 ± 3.42%, which was significantly higher than that of VoNP (23.63 ± 0.96%). These data suggest that the VoNPs significantly slowed down the release of Vo protein in vitro. To characterize the release of protein from nanoparticles in vivo, green fluorescent protein (GFP) was encapsulated in nanoparticles (GFP-NPs) and intramuscularly administered to nude mice. Figure 6b shows the images of mice at 0.5, 1, 2, 4, 8, 12, 24, 36 and 48 h after the administration. The tissue fluorescence of mice administered GFP-NPs decreased much slower than that of mice administered soluble GFP. Moreover, the quantification of fluorescence intensity confirmed this observation (Fig. 6c). These results suggest that our nanoparticles can slow the release of encapsulated proteins, which may improve the in vivo stability of therapeutics.

### Fresh VoNPs elicited protective immunity in mice

**Fig. 7:**
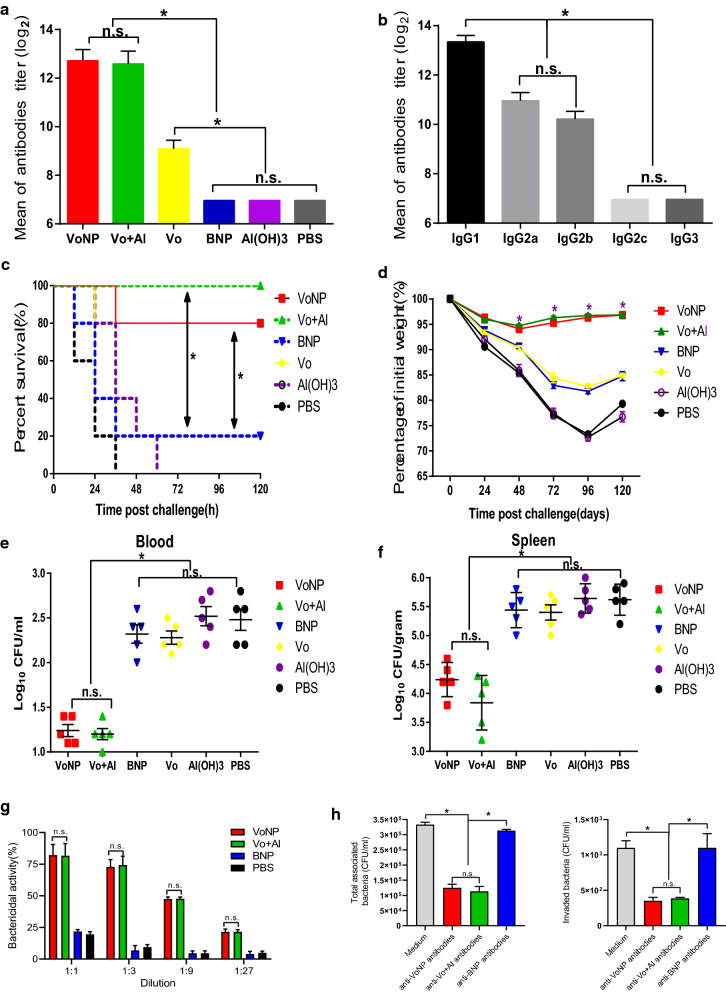
Fresh VoNPs elicited protective immunity in mice. **a** The bar represents the titers of the anti-Vo IgGs in the mice after vaccination with fresh VoNPs, Vo formulated with Al(OH)_3_ as a conventional adjuvant (Vo + Al), or soluble Vo alone. **b** The bar represents the titers of the anti-Vo IgG subtypes in the mice vaccinated with VoNPs. **c** Mice immunized with VoNPs, Vo + Al or Vo alone. The bar represents the survival of the mice after challenge with a lethal dose of *E. coli* K1 RS218. **d** The bar represents the percentage of initial body weight of the mice after challenge with a sublethal dose of *E. coli* K1 RS218. **e** The bar represents the number of bacteria per ml blood 48 h after challenge with a sublethal dose of *E. coli* K1 RS218. **f** The bar represents the number of bacteria per gram of spleen tissue 48 h after challenge with a sublethal dose of *E. coli* K1 RS218. BNP indicates the blank nanoparticle control. **g** Opsonophagocytic killing assay of anti-VoNP serum. The bar represents the percentage of killed bacteria in a series of dilutions. **h** The effects of anti-VoNP antibodies on bacterial attachment and invasion. The bar represents the mean value and the standard error of the number of total associated bacteria (left panel) and invading bacteria (right panel). *n.s.* no significant difference; *P < 0.05

BALB/c mice were immunized with fresh VoNPs, and the titer of total anti-Vo IgG in sera was detected. As shown in Fig. 7a, the titer of the anti-Vo antibodies in the VoNP group was significantly higher than that of the BNP group, Al(OH)_3_ group and PBS group. However, no significant difference in titer was observed between the VoNP and Al(OH)_3_-formulated Vo groups (*P* > 0 05), which suggested that the adjuvant effect of CS-PLGA nanoparticles was similar to that of Al(OH)_3_. In addition, the subtypes of Vo-specific IgG antibodies were determined. The results showed that the titer of IgG1 was significantly different from that of IgG2a, IgG2, IgG2c and IgG3 (*P* < 0. 05). These results indicated that a Th2-predominant response was elicited (Fig. 7b).

Then, an acute *E. coli* K1-induced mouse model of bacteriemia was used to evaluate the protective effects of the VoNPs. Figure 7c shows the survival of the mice after challenge with a lethal dose of *E. coli* K1 (1 × 10^8^ CFU/mouse). All the mice in the Al(OH)_3_ group and the PBS group died within 60 h and 36 h, respectively, and the survival rates of the BNP group and the Vo protein group were only 20%, with no significant difference in survival between the two groups. The survival rates of the VoNP group and the Vo plus Al(OH)_3_ group were 80% and 100%, respectively, which were significantly higher than those of the other groups (*P* < 0 05). Interestingly, there was no significant difference in survival between the VoNP group and the Vo plus Al(OH)_3_ group.

To further understand the mechanism of VoNP-induced protection, we challenged the immunized mice with a sublethal dose of *E. coli* K1 (1 × 107 CFU/mouse). The body weights of the BNP, Vo, Al(OH)_3_ and PBS groups decreased significantly and began to recover 96 h after the challenge. The decrease in the VoNP group and the Vo plus Al(OH)_3_ group was lower than that in the other four groups and began to recover 48 h after the challenge (Fig. 7d). Moreover, the numbers of bacteria in the blood and spleens of the mice in the VoNP group and the Vo + Al(OH)_3_ group were significantly lower than those in the other four groups (*P* < 0 05). However, there was no significant difference between the VoNP group and the Vo + Al(OH)_3_ group (*P* > 0 05) (Fig. 7e, f). Collectively, the above results showed that fresh VoNPs could elicit protective immunity, which was equivalent to that of the Vo vaccine formulated with Al(OH)_3_.

Additionally, the in vitro protective effect of anti-VoNP antibodies was evaluated. Results of the opsonophagocytic killing assay showed that the killing activity of antisera from VoNP-immunized animals was as efficient as that of the antisera elicited using Vo formulated with conventional Al(OH)_3_ (Fig. 7g). By contrast, no apparent killing was observed using the sera of mice immunized with BNP. Moreover, the anti-VoNP antibodies significantly inhibited the attachment and invasion of *E. coli* K1, and the effect was as potent as that of anti-Vo antibodies elicited using Al(OH)_3_ as an adjuvant (Fig. 7h). In sharp contrast, no significant effect of anti-BNP antibodies was observed. Taken together, the anti-VoNP antibodies exert a protective effect in vitro by inducing opsonophagocytic killing as well as blocking bacterial attachment and invasion.

### VoNPs could still elicit protective immunity in mice after 180 days of storage

**Fig. 8:**
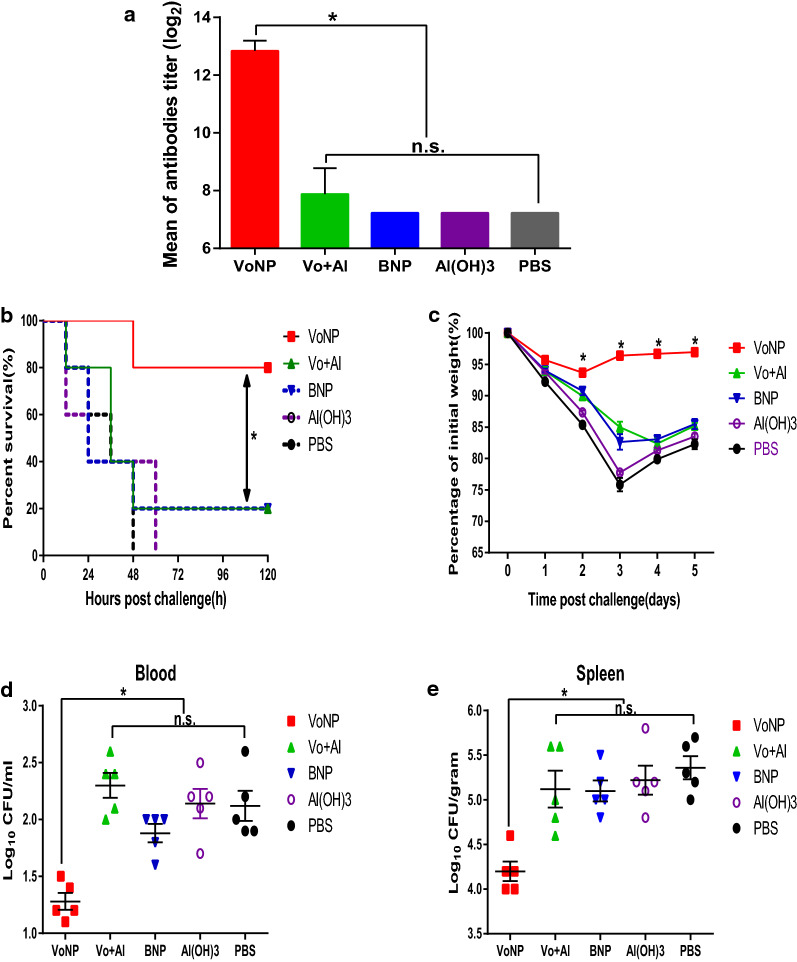
VoNPs still elicited protective immunity in mice after 180 days of storage. **a** The bar represents the titers of the anti-Vo IgGs in the mice immunized with the VoNPs and Vo  + Al, which were stored at 4 °C for 180 days. **b** The bar represents the survival of the mice after challenge with a lethal dose of *E. coli* K1 RS218. Before the challenge, the mice were immunized with the VoNPs or Vo + Al aliquots that had been stored at 4 °C for 180 days. **c** Mice were immunized with VoNPs or Vo + Al, which were stored at 4 °C for 180 days. The bar represents the body weight of the mice after challenge with a sublethal dose of *E. coli* K1 RS218. **d** The bar represents the number of bacteria per ml blood 48 h after a sublethal challenge with *E. coli K1* RS218. **e** The bar represents the number of bacteria per gram of spleen tissue 48 h after a sublethal challenge with *E. coli K1* RS218. BNP indicates the blank nanoparticle control. *n.s.* no significant difference; *P < 0.05

To evaluate the immunogenic stability of the VoNPs, we immunized the mice with VoNPs and Vo adsorbed with Al(OH)_3_ proteins after being stored at 4 °C for 180 days. As shown in Fig. 8a, there was no significant difference in the anti-Vo titers among the Vo adsorbed with Al(OH)_3_ and the BNP, Al(OH)_3_ and PBS groups (*P* > 0 05), which indicated the reduction of the humoral immune response of Vo formulated with Al(OH)_3_ (Fig. 8a). However, long-term immunization with preserved VoNPs could induce significant high-level anti-Vo IgG antibodies as before. Next, the immune protective effect of preserved VoNPs was evaluated. All the mice in the Al(OH)_3_ adjuvant group and the PBS group died at 60 h and 48 h, respectively. Surprisingly, the survival rate of the long-term preserved Vo plus Al(OH)_3_ group was only 20%, which was significantly lower than that of the fresh Vo plus Al(OH)_3_ group, indicating that this formulation was not stable (Fig. 8b). However, the survival rate of the VoNP group remained at 80%, which was significantly higher than that of the other groups (*P* < 0.05). As expected, similar trends in body weight change and the number of colonized bacteria were observed. The VoNP-immunized mice showed the lowest body weight loss and colonized *E. coli* K1 in the blood and spleens compared to the mice immunized with Vo plus Al(OH)_3_, BNP, Al(OH)_3_ and PBS (Fig. 8c–e). Collectively, these data suggested that immunization of the VoNPs after 180 days of storage could still protect against *E. coli K1* bacteremia in mice.

## Discussion

Antibiotics are an effective tool for treating neonatal meningitis caused by *E. coli* K1, but their effectiveness is limited by the increasing emergence of drug resistance. In particular, the emergence of a number of drug-resistant epidemic strains has exacerbated the challenge of treating *E. coli* K1 infections [18, 19]. Therefore, vaccines are an alternative solution to this urgent problem. Capsular polysaccharides (CPs) are the main virulence factors of bacteria and are therefore also used as primary antigen candidates for vaccines such as the *Streptococcus pneumonia* vaccine and the *Haemophilus influenzae* vaccine. However, the CPs (2→8)-α-Neu5Ac of *E. coli* K1 cross-react with host normal tissues, posing a potential risk of a pathological immune response [20]. Thus, protein antigens are used as alternative candidate antigens. OmpA is the major antigen of *E. coli* K1 and is involved in key bacterial pathogenic processes [5]. Our results showed that Vo and VoNPs conferred considerable protection by targeting OmpA [12], which suggested that protein vaccines are likely one direction for successful *E. coli* K1 vaccines. In recent years, many pathogenic factors, such as Ibe, AslA, and NlpI, have been identified [21]. These proteins are also important vaccine targets and need further investigation in the future.

Epitope-based vaccines have many unique advantages, such as precise immune responses, avoidance of antibody-dependent enhancement (ADE), and flexibility of multiepitope combinations [22, 23]. With the help of bioinformatics, immunoproteomics and other methods, many epitopes or immunologically dominant domains have been identified. However, their applications in epitope-based or multiepitope vaccines are unsatisfactory [24, 25]. One primary reason is that the antigens are usually unstable and have poor bioavailability because of their unnatural structure and low molecular weight. To solve the problem of epitope-based vaccines, researchers have used lipopeptides, branching polypeptides, unlipidated polypeptides, VLPs, polymer nanoparticles, epitope grafts and other strategies [26, 27]. In this study, we found that CS-PLGA-coated nanoparticles could significantly maintain the long-term stability and immunogenicity of Vo. These results provide more evidence for the usage of CS-PLGA and other natural polymers as vaccine delivery vehicles.

One interesting finding is that the level of humoral response induced by the VoNPs was as efficient as that with aluminum hydroxide adjuvant. There are three possible explanations. First, antigens in the PLGA nanoparticles may be released slowly during the process of polymer lysis and degradation, which could continuously stimulate the immune system [28]. Second, PLGA nanoparticles may be more easily recognized and processed by antigen-presenting cells due to their virus-like granular structure [29]. Third, the modification of chitosan could enhance both of the above two processes [30]. To date, nasal immunization of vaccines delivered by PLGA nanoparticles can induce strong mucosal immune responses, such as the production of sIgA [31]. In the future, we plan to change the immune routine to improve the immune response and protection of VoNPs.

It is interesting to note that the zeta potential of VoNPs increased significantly after the freeze-drying. It is well known that PLGA-NP has a negative surface charge due to its polymeric matrix [32], while CS nanoparticles have a markedly positive potential [33]. In this study, we produced chitosan-modified PLGA nanoparticles using a double emulsion-solvent evaporation method, which relies on physical modification [34]. Thus, it is inevitable that a certain amount of free CS remained in the VoNPs. The free hydrophilic CS may have re-coated on the surface of VoNPs after the freeze-drying and rehydration processes. Consequently, the zeta potential of VoNPs became nearly neutral (0.6110 ± 0.0267 mV) after the freeze-drying process.

One limitation of our study is that the immune protective effect of VoNPs was not evaluated in an *E. coli* K1 meningitis model. Currently, there are two ways to induce *E. coli* K1 meningitis in mice. One way is to intraperitoneally inject newborn mice or rats (~ 3 days old) with *E. coli* K1 [35, 36]. The bacteria enter the brain through the immature barrier and cause meningitis. The other way is to directly inject *E. coli* K1 into the ventricles of the brain and cause meningitis [37]. These two models can be used for the investigation of the pathogenesis of *E. coli* K1 and the evaluation of other therapeutic drug effects but are not suitable for the evaluation of VoNPs and other *E. coli* K1 vaccines. Because the immunization of VoNPs requires at least 21 days, mice at this stage can no longer develop meningitis by intraperitoneal injection. In addition, due to the existence of the blood–brain barrier, it is difficult for immune substances to enter the brain tissue and show effects, and thus, it is not possible to evaluate the vaccine’s effect by injecting bacteria directly into the brain ventricles. The next step is to induce meningitis in other more susceptible animals to address this issue.

In summary, we successfully generated VoNPs and found that a freeze-drying procedure increases the stability of the VoNPs without changing the shape, size distribution and encapsulation. The VoNPs were immunoprotective in mice even after storage for as long as 180 days, which is far better than the aluminum adjuvant. Collectively, we found an effective strategy to improve the stability of Vo to maintain its immunogenicity, which will promote future development of vaccines against *E. coli* K1.

## Data Availability

The datasets used and/or analyzed during the current study available from the corresponding author on reasonable request.
